# Using an Untargeted Metabolomics Approach to Identify Salivary Metabolites in Women with Breast Cancer

**DOI:** 10.3390/metabo10120506

**Published:** 2020-12-10

**Authors:** Daniele Xavier Assad, Ana Carolina Acevedo, Elisa Cançado Porto Mascarenhas, Ana Gabriela Costa Normando, Valérie Pichon, Helene Chardin, Eliete Neves Silva Guerra, Audrey Combes

**Affiliations:** 1 Laboratory of Oral Histopathology, Health Sciences Faculty, University of Brasília Campus Universitário Darcy Ribeiro, Brasília DF 70910-900, Brazil; dxassad@gmail.com (D.X.A.); acevpoppe@gmail.com (A.C.A.); elisaporto@gmail.com (E.C.P.M.); gabinormando@gmail.com (A.G.C.N.); elieteneves@unb.br (E.N.S.G.); 2 Medical Oncology Department, Hospital Sírio-Libanês, SGAS 613 Conj. E Bl. B, Brasília DF 70200-730, Brazil; 3 Medical Oncology Department, Cettro—Centro de Câncer de Brasília, SMH/N Quadra 02, 12 Andar, Brasilia DF 70710-904, Brazil; 4 Department of Analytical, Bioanalytical Sciences and Miniaturization (LSABM), UMR CBI 8231, ESPCI Paris, CNRS, PSL University, 75005 Paris, Ide de France, France; valerie.pichon@espci.fr (V.P.); helene.chardin@espci.fr (H.C.); 5 Campus UPMC, Sorbonne Université, 75005 Paris, France; 6 Faculté de Chirurgie Dentaire, Université de Paris, 92120 Montouge, France

**Keywords:** metabolites, breast cancer, biomarkers, METLIN database, RPLC/MS analysis

## Abstract

Metabolic alterations are a hallmark of the malignant transformation in cancer cells, which is characterized by multiple changes in metabolic pathways that are linked to macromolecule synthesis. This study aimed to explore whether salivary metabolites could help discriminate between breast cancer patients and healthy controls. Saliva samples from 23 breast cancer patients and 35 healthy controls were subjected to untargeted metabolomics using liquid chromatography-quadrupole time-of-flight mass spectrometry and a bioinformatics tool (XCMS Online), which revealed 534 compounds, characterized by their retention time in reverse-phase liquid chromatography and by the *m*/*z* ratio detected, that were shared by the two groups. Using the METLIN database, 31 compounds that were upregulated in the breast cancer group (*p* < 0.05) were identified, including seven oligopeptides and six glycerophospholipids (PG14:2, PA32:1, PS28:0, PS40:6, PI31:1, and PI38:7). In addition, pre-treatment and post-treatment saliva samples were analyzed for 10 patients who experienced at least a partial response to their treatment. In these patients, three peptides and PG14:2 were upregulated before but not after treatment. The area under the curve, sensitivity, and specificity for PG14:2 was 0.7329, 65.22%, and 77.14%, respectively. These results provide new information regarding the salivary metabolite profiles of breast cancer patients, which may be useful biomarkers.

## 1. Introduction

Metabolic alteration is a hallmark of cancer cells and their malignant transformation is characterized by multiple changes in metabolic pathways that are linked to macromolecule synthesis [1,2]. Thus, cancer cells have altered metabolic requirements to facilitate inappropriate proliferation and survival, and these cells must simultaneously coordinate nutrient uptake and metabolism to meet their catabolic and anabolic demands. The classic example of a reprogrammed metabolic pathway in cancer is the Warburg effect or “aerobic glycolysis” [3]. Breast cancer (BC) is one of the most commonly diagnosed cancers and is the leading cause of cancer-related mortality among women [4]. The diagnosis of BC involves (i) identification of a suspected lesion via radiological screening and (ii) a confirmatory biopsy [5]. However, conventional screenings with physical examinations and a mammography provide less-than-desirable sensitivity (54%) and specificity (77%) [6] and, despite its invasiveness and risk of morbidity, breast biopsy with histopathological evaluation remains the gold standard diagnostic method [7]. Thus, the identification of specific and sensitive BC biomarkers is an important goal to improve BC diagnosis, treatment monitoring, and patient comfort.

Several studies have developed targeted and untargeted metabolomics approaches to highlight altered metabolites (up or down regulated) associated with BC and the most commonly used biological samples are biopsies, plasma, or serum [8], although a few studies also used urine or saliva [8]. Indeed, saliva reflects the body’s physiological condition and may be useful for monitoring a patient’s clinical status and identifying systemic diseases [9]. Saliva has already been widely used in genetic testing because of its more favorable transport stability relative to that of blood [10]. Saliva is 99% water but also contains mucus, electrolytes (such as K^+^ or Na^+^) [11], nucleic acids, and proteins [9]. Relative to blood-based testing, saliva-based testing is simpler, easier, and safer, given the non-invasive method of collection [10].

In 2010, Sugimoto et al. [12] described salivary metabolic profiles for oral, breast, and pancreatic cancers based on an untargeted metabolomic approach using capillary electrophoresis coupled with a time-of-flight mass spectrometer, which identified upregulation of 14 amino acids (AAs) (including taurine and lysine). In addition, a review of salivary biomarkers for diagnosing BC revealed that several targeted approaches highlighted sialic acid, taurine, proline, and valine as potential diagnostic values [13]. Three metabolomic studies have also indicated that the saliva of Asian BC patients contained elevated levels of various AAs [12,14,15].

The development of bioinformatic tools has made it relatively simple to automate the highlighting of distinct metabolite features from different groups of samples [16]. For example, the XCMS Online tool detects and identifies chromatographic features with varying relative intensity values for comparisons between sample groups, with the reported data including the *p*-values and fold changes [17,18]. These tools have potential utility for identifying early subclinical markers that can be used to predict the development of BC and facilitate early intervention. Therefore, the goal of the present study was to identify salivary metabolites that were differentially expressed in the saliva of BC patients and healthy controls and might guide early diagnoses and/or treatment regardless of the type and stage of cancer. For this purpose, an untargeted metabolomics approach using liquid chromatography-quadrupole time-of-flight mass spectrometry and a bioinformatics tool was applied to saliva samples of healthy controls, patients with BC, and ten patients with BC who received treatment that has been shown to be effective.

## 2. Results

### 2.1. Patient Characteristics

This study included 23 women with BC (mean age: 47.52 ± 9.79 years) and 35 healthy women from the general population (mean age: 42.00 ± 13.83 years). All BC cases involved invasive ductal carcinoma, although one case also involved a micropapillary component and another case also involved squamous differentiation. None of the controls had a history of cancer treatment. Table 1 shows the demographic characteristics of the 50 subjects who provided saliva samples. There were no significant differences regarding age, menopause status, tobacco use, medication use, or childbearing. Table 2 shows the BC patients’ clinicopathological characteristics. A complete list of subject characteristics is provided in Supplementary Table S1.

### 2.2. Quality Control for Untargeted Metabolomics

The conditions of the stored saliva samples were evaluated at different points throughout the −80 °C storage period (6 months). Each sample was injected twice into the liquid chromatography (LC)/mass spectrometry (MS) system, and the same LC/MS profiles were obtained at all time points, which suggested appropriate storage conditions and reproducible analytical methods. In order to validate the procedure for the saliva collection and storage, the presence of previously described cancer-related salivary metabolites (AAs including lysine and taurine) was checked manually in LC/MS chromatograms (via search of the corresponding *m*/*z* ratio) (Supplementary Table S2) [12,18,19,20,21,22,23,24,25,26,27,28], although no significant inter-group differences were observed using XCMS Online profile comparison (all *p* > 0.05).

### 2.3. The LC/MS Profiles of the Healthy Controls and BC Patients

The LC/MS salivary profiles were compared between BC patients and healthy controls using XCMS Online, which revealed 534 compounds that were present in both groups (evidenced by the same *m*/*z* ratio during MS analysis and same retention time during reverse phase liquid chromatography). The resulting LC/MS profiles are provided in Supplementary Figure S1. Significant inter-group differences were observed for 37 compounds (upregulated or downregulated, *p* < 0.05 for all). Only one downregulated compound was ignored because we chose to focus on upregulated metabolites in the BC patients. Then, the extracted ion chromatograms (EICs) for the 36 corresponding compounds were manually evaluated, it was confirmed that 35 compounds were upregulated, and 1 compound was excluded because it did not correspond to a chromatographic peak. The molecular weights of all known medications used by the subjects were compared with those of each upregulated compound (according to the determined charge state in the MS spectrum), which confirmed that the identified compounds were not prescribed medications or their metabolites (Supplementary Table S3). The proposed metabolite names were also search in the METLIN database, which identified 4 of the 35 compounds as potentially being drugs or phytochemical compounds. These compounds corresponded to dioscin (an antifungal agent) or donepezil (an oral medication used to treat Alzheimer’s disease), desglucomusennin (a phytochemical compound), dilazep (a vasodilator), and tetrahydrogambogic acid (a compound isolated from fruits) (Table 3, lines 1–4). Among the 31 remaining compounds, the METLIN database proposed a putative identification for 13 metabolites (seven oligopeptides and six glycerophospholipids) based on the experimental *m*/*z* and charge state (Table 3, lines 5–17). The characteristics of the 18 unidentified upregulated compounds (i.e., retention time, fold change between the patient and control groups, and *p*-value) are shown in Supplementary Table S4.

### 2.4. Comparing the LC/MS Profiles before and after Systemic Treatments

**Figure 1:**
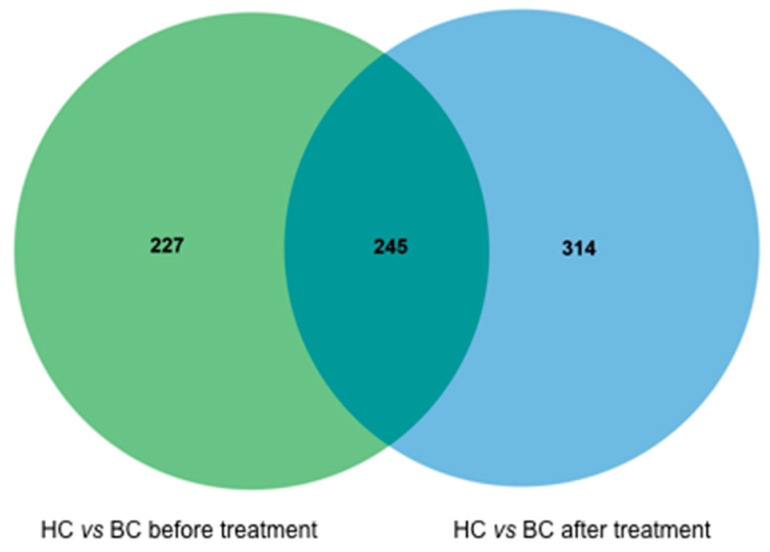
Venn diagrams comparing the liquid chromatography/mass spectrometry (LC/MS) profiles before and after treatment. HC corresponds to healthy control and BC to Breast cancer patients.

Saliva samples from 10 patients receiving a systemic treatment (eight cases involving neo-adjuvant chemotherapy and two cases involving endocrine chemotherapy) were analyzed before and after treatment administration to identify any differences in the LC/MS profiles. Overlay of LC/MS profiles are shown in Supplementary Figure S2. All 10 patients had at least a partial response, therefore a change in the LC/MS profile could suggest a treatment response biomarker. The patients’ characteristics, treatments, and responses are listed in Supplementary Table S5. The LC/MS profiles of the control group were also compared to those of the patients’ pre-treatment and post-treatment groups (Figure 1). The 227 compounds with altered pre-treatment regulation were searched for the 13 previously identified compounds, which confirmed that four of the identified compounds were upregulated before treatment and returned to healthy control level after treatment. These compounds with pre-treatment upregulation corresponded to ions with *m*/*z* ratios of 440.23 (H-Arg-Arg-Ser-OH), 533.22 (PG14:2), 543.23 (H-Ala-Lys-Phe-Trp-OH or H-Gly-Lys-Thr-Ser-OH or H-Arg-Arg-Ser-Ser-OH), and 630.33 (H-Phe-Lys-Lys-Trp-OH or H-Phe-Gln-Arg-Tyr-OH).

The green circle represents all metabolites found in pair-wise comparisons of the 35 healthy controls (HCs) and 10 breast cancer patients (BCs) before treatment. The blue circle represents all metabolites found in pair-wise comparisons of the 35 HCs and 10 BCs after the treatment. A total of 245 compounds found in the BC group did not change when the results before and after treatment were compared. A total of 227 compounds were upregulated or downregulated only before the treatment and were not present after treatment, and 314 compounds were only present after treatment.

### 2.5. The Receiver Operating Characteristic Curve Analysis

The diagnostic test accuracy measurements for salivary metabolites are shown in Table 4. ROC curves were used to evaluate the predictive value of each compound, based on the optimal cut-off values for the salivary compounds and their sensitivity and specificity values (Table 4). Among the six identified lipids, the only significant difference in the area under the curve (AUC) values was observed between PG14:2 and PA32:1 (*p* = 0.0434). Among the lipids, the highest AUC value was 0.7329 (PG14:2), the highest sensitivity was 65.22% (PG14:2), and the highest specificity was 88.57% (PS28:2). Among the peptides, the highest AUC value was 0.7478 (H-Phe-Phe-Gln-Trp-OH), the highest sensitivity was 73.91% (H-His-Lys-[Ala-Ser]-OH or [Gly-Thr]-OH), H-Phe-Phe-Gln-Trp-OH, and H-Arg-Arg-Ser-OH), and the highest specificity was 80% (H-Glu-Phe-Gln-Arg-OH or H-Ile-Lys-Gln-Trp-OH). Among the unidentified compounds, the highest AUC value was observed for a 614.82 *m*/*z* compound, the highest sensitivity (86.96%) was observed for a 594.82 *m*/*z* compound, and the highest specificity (82.86%) was observed for a 356.69 *m*/*z* compound.

## 3. Discussion

This study explored whether salivary metabolites could be detected using an untargeted approach and help discriminate between BC patients and healthy control subjects. Some constituents can be found in both blood and saliva, although saliva is a less expensive, simpler, and non-invasive diagnostic material [29]. Substances can pass through the epithelial membranes via several mechanisms, including passive diffusion (for highly lipid-soluble molecules), an active process (for electrolytes, IgA), and ultrafiltration through membrane pores (for small polar molecules, <300 Da) [30]. We analyzed biomolecules in the patients’ saliva using LC coupled with an MS analyzer, equipped with an electrospray source, to determine the *m*/*z* ratios of the ions. The full mass spectrums were then analyzed using a bioinformatics tool (XCMS Online), which identified 31 compounds that were upregulated in BC patients and 13 potentially relevant compounds (seven oligopeptides and six lipids).

Relative to normal cells, tumor cells dramatically alter AA uptake and secretion, which accounts for the majority of the carbon-based biomass production in rapidly proliferating cancer cells [31]. In addition, AAs also contain nitrogen and are the dominant nitrogen source for hexosamines, nucleotides, and other nitrogenous compounds in rapidly proliferating cells [32]. Cheng et al. [31] used a targeted approach to evaluate specimens from 27 BC patients, and reported that the BC patients had higher salivary levels of 17 AAs [14]. Sugimoto et al. used an untargeted approach and identified 28 salivary metabolites, including 14 AAs that were upregulated in BC patients (all *p* < 0.05) [12]. However, they did not perform direct identification, and no significant differences were observed for these metabolites when the BC patients were compared to the patients with other cancers (oral and/or pancreatic cancer). Zhong et al. [33] used an untargeted approach (30 BC patients and 25 healthy controls) to identify 18 metabolites, including phenylalanine, citrulline, and histidine, which were confirmed using standard samples. In the present study, these AAs were present in the samples, but not statistically significantly upregulated in BC patients. However, the seven oligopeptides upregulated in our BC patients were composed of 14 AAs that were included in those identified by Cheng et al. Thus, because saliva contains peptidases and proteinases, some differences in collection and/or storage protocols may influence its final free amino acid composition. Moreover, given that our study evaluated Brazilian patients, and that previous studies evaluated Asian patients, ethnicity-related factors might have contributed to these differences [32,34].

The de novo biosynthesis of fatty acids is low in normal adult tissues, although tumorigenesis is associated with a dramatic increase in lipid production [35], which has also been confirmed in BC patients [15]. Phospholipids are an essential component of the cell membrane and are involved in a variety of biological functions, such as division of the cytoplasm, inter-cell adhesion, and protein storage [36]. Our results identified six glycerophospholipids that might be related to BC (PG14:2, PA32:1, PS28:0, PS40:6, PI31:1, and PI38:7). Based on the Human Metabolome Database, PG14:2, PS28:0, PS40:6, and PI31:1 have extracellular and membrane localizations, while PA32:1 has no cellular localization. The pathways of the identified ions are listed in Table 5.

The PG14:2 phosphatidylglycerol has a phosphoglycerol moiety occupying a glycerol substitution site and is related to glycerophospholipid metabolism [37]. The PA32:1 phosphatidic acid is a glycerophosphate with a phosphate moiety occupying a glycerol substitution site [38] and is extremely important as an intermediate in the biosynthesis of triacylglycerols and phospholipids, which are related to the cardiolipin biosynthesis pathway [39]. Cardiolipin is an important component of the inner mitochondrial membrane, where it accounts for approximately 20% of the total lipid composition [38] and is essential for the optimal functioning of numerous enzymes that are involved in mitochondrial energy metabolism, as well as triacylglycerol biosynthesis [40]. The PS28:0 and PS40:6 phosphatidylserines have a phosphorylserine moiety occupying a glycerol substitution site and are related to phosphatidylcholine biosynthesis, phosphatidylethanolamine biosynthesis, glycerophospholipid metabolism, and the lipid metabolism pathway [37]. The PI31:1 and PI38:7 phosphatidylinositols are key membrane constituents and participate in essential metabolic processes, both directly and indirectly via a number of metabolites [37]. The PI38:7 phosphatidylinositol is related to lysolipid incorporation into the ER pathway, phosphatidylcholine biosynthesis, phosphatidylethanolamine biosynthesis, glycerophospholipid metabolism, and the lipid metabolism pathway [37].

Zhong et al. [33] identified salivary lipids that were significantly elevated in BC patients [15], including three lipids that were related to phosphatidylcholine biosynthesis (PE22:0/20:4, PC18:1/16:0, PS14:1/16:1) and two lipids that were related to glycerophospholipid metabolism (LysoPE18:2/0:0 and LysoPC22:6). The present study also revealed alterations in these metabolic pathways, based on increases that were observed for PS28:0, PS40:6, PI38:7, PA32:1, PS28:0, PS40:6, PI38:7, and PG14:2. However, our ROC curve analysis revealed no significant differences in the metabolites’ AUCs, with the exception that PG14:2 was superior to PA32:1 (*p* = 0.0434), and the highest AUC values were observed for PG14:2 (0.7329) and PI38:7 (0.6609). Zhong et al. [33] also identified high AUC values for predicting BC using three upregulated lipids, which were LysoPC18:1 (AUC: 0.92), LysoPC22:6 (AUC: 0.92), and MG 0:0/14:0/0:0 (AUC: 0.929). These higher AUC values were clearly superior to the values for the lipids that we identified in the present study. However, the present study compared salivary compounds before and after treatment in 10 patients with good treatment response and found that four compounds (three peptides and PG14:2) were upregulated before treatment but subsequently normalized after treatment. Thus, even if this sub-analysis was clearly limited by the small sample size, ours is the first report to examine the effects of systemic cancer treatment on salivary metabolite profiles, which may suggest that salivary metabolites could be useful for monitoring treatment response.

In conclusion, the present study identified 31 upregulated compounds in saliva of BC patients, and we were able to identify 13 potentially relevant compounds via the METLIN database (seven peptides and six lipids). A comparison of pre-treatment and post-treatment metabolite profiles from 10 patients revealed that three peptides and PG14:2 were upregulated before treatment and returned to normal levels after relatively successful treatment. Thus, although caution must be exercised when interpreting this finding, it may be interesting to follow these compounds specifically to monitor a response to treatment of breast cancer patients; these metabolites could be useful biomarkers for BC treatment response. The ROC curve analyses revealed that the salivary lipids provided good specificity and fair sensitivity for identifying BC, although a larger cohort of BC patients and healthy controls is needed to confirm our findings. Nevertheless, we believe that our results indicate that salivary testing may be useful for the early diagnosis of BC.

## 4. Materials and Methods

### 4.1. Ethical Considerations

This prospective study was conducted in accordance with the principles of the Declaration of Helsinki, and the study protocol was approved by the Research Ethics Committee of the Faculty of Health Sciences, University of Brasilia (Plataforma Brasil protocol: 57449716.5.0000.0030). All individuals provided written informed consent before participating in the study.

### 4.2. Study Participants

The control subjects were healthy women who were recruited from the general population to undergo a normal physical examination and radiological breast imaging. The inclusion criteria for the control subjects were women with normal clinical and imaging findings, and the exclusion criterion was abnormal imaging or clinical findings. The controls were selected to have ages that matched the ages of the cases. Consecutive BC patients were recruited at the Hospital Universitário de Brasília, Hospital de Base do Distrito Federal, Hospital Sírio-Libanês, and Centro de Câncer de Brasília-Cettro between October 2016 and October 2017. The inclusion criteria for the BC group were: (i) not pregnant or lactating; (ii) no active oral/dental disease; (iii) no prior neoplasia, except for non-melanomatous skin cancers, cervical carcinoma in situ, or benign tumors (e.g., adenomas); (iv) no impaired renal function, congestive heart failure, or active infection (e.g., hepatitis and HIV); and (v) a histopathological diagnosis of BC. These patients were enrolled before any systemic treatment (i.e., neo-adjuvant chemotherapy or palliative endocrine/chemotherapy) and before definitive surgery. There was no central pathological review for the present study. Tumor staging was performed based on the 7th edition of the American Joint Committee on Cancer guidelines [41]. Molecular profile classification was performed according to the Saint Gallen consensus [42]: (i) luminal A-like: all of estrogen receptor (ER)-positive, progesterone receptor (PgR)-positive, human epidermal growth factor receptor 2 (HER2)-negative, and Ki-67 ‘low’; (ii) luminal B-like HER2-negative: ER-positive, HER2-negative, and at least one of Ki-67 ‘high’ and PgR-negative or PgR-low; (iii) luminal B-like HER2-positive: ER-positive, HER2 over-expressed or amplified, any Ki-67, and any PgR; (iv) HER2-positive (non-luminal): HER2 over-expressed or amplified, ER-negative, and PgR-negative; and (v) triple-negative: ER-negative, PgR-negative, and HER2-negative. Stage I–III cases were treated using neo-adjuvant chemotherapy with or without double HER2 blockade, followed by surgery with or without radiotherapy, and with or without adjuvant endocrine treatment. Stage IV cases were treated using palliative chemotherapy or endocrine therapy with or without double HER2 blockade.

### 4.3. Specimen Collection, Transportation, and Preparation

Stimulated saliva samples were collected from each participant, who abstained from eating, drinking, smoking, and performing oral hygiene procedures for more than 1 h before sample collection. The participants were instructed to chew a cotton swab (Salivette^®^; Sarstedt AG & Co., Nümbrecht, Oberbergischer Kreis, Germany) for 2 min, which was then placed in a plastic container and packaged in a Styrofoam box with recyclable ice packets for less than 4 h before transport and processing. The Salivette^®^ provides an optimal method for hygienic collection of saliva. Moreover, the dimensionally stable and biocompatible synthetic swab stands out for its superior absorption quality and virtually complete saliva recovery under the recommended centrifugation conditions. The saliva sample (typically 5–10 mL) was centrifuged for 5 min at 3000 rpm and 8 °C. After centrifugation, a sediment was observed, and the supernatant was transferred to a clean Eppendorf tube and frozen at −80 °C until further analysis. Before liquid chromatography (LC) and mass spectrometry (MS) analysis, the saliva samples were diluted with one volume of the mobile LC phase (90:10, water with 0.1% formic acid (*v*/*v*)/acetonitrile with 0.1% formic acid (*v*/*v*), *v*/*v*). High-performance liquid chromatography (grade acetonitrile and formic acid were supplied by CarloErba (Val de Reuil, France), and ultra-pure water was obtained using a Milli-Q Purification system (Millipore, Molsheim, France).

### 4.4. Liquid Chromatography-Quadrupole Time-of-Flight Mass Spectrometry (LC-Q-TOF/MS)

The LC/MS analyses were performed using an Agilent 1100 LC system (Agilent Technologies, Les Ulis, France) coupled with a MicrOTOF-Q II Mass Spectrometer (Bruker Daltonics, Wissem-bourg, France) with an electrospray ionization (ESI) source. The separation was performed using an Atlantis dC18 column heated at 40 °C (150 × 2.1 mm, 3 µm, 100 Ǻ; Waters Corporation, Milford). The mobile phase was composed of (A) water with 0.1% formic acid (*v*/*v*) and (B) acetonitrile with 0.1% formic acid (*v*/*v*). The elution gradient started at 10% B, which was increased to 90% B over a 45-min interval, with a 2-min plateau, a 3-min return to the initial composition, and a 10-min final equilibration step. The flow rate was set at 0.2 mL/min, the injection volume was 20 µL, and each sample was injected twice. The ESI–TOF–MS analysis was performed in positive ionization mode, and the mass-detection range was set at *m*/*z* 50–1200. The ESI source parameters were a drying gas (N_2_) flowrate of 5.0 L/min, drying gas temperature of 200 °C, nebulizing gas pressure of 10 psi, and capillary voltage of 4500 V. The ion transfer method used two different settings: a couple collision RF/transfer time equal to (i) 100 Vpp/23 µs during 30% of the acquisition time (300 µs) and (ii) 400 Vpp/100 µs during 70% of the acquisition time (700 µs) (i.e., an acquisition time of 1 s for each MS spectrum). The collision energy and the pre-pulse storage were maintained at 5 eV and 5 µs, respectively. All data acquisitions were controlled using TOF Control software (version 3.4, BrukerDaltonics, Wissembourg, France), and Hystar software (version 3.2, Bruker Daltonics, Wissembourg, France) was used to interface the HPLC and MS systems.

### 4.5. Metabolic Pathways

The metabolites identified using XCMS Online were searched for in the Human Metabolome Database (www.hmdb.ca), KEGG network (www.genome.jp), PubChem (www.pubchem.ncbi.nlm.nih.gov), Small Molecule Pathway Database (www.smpdb.ca), and LIPID MAPS Lipidomics Gateway (www.lipidmaps.org).

### 4.6. Data Analysis

Subject characteristics were compared using the Student’s *t*-test, the Chi-squared test, or Fisher’s exact test. Differences were considered significant at *p*-values < 0.05. Receiver operating characteristic (ROC) curves were used to evaluate the predictive value of each biomarker based on the methods of DeLong et al. [43]. Optimal cut-off points on the ROC curves were identified based on: (i) the shortest Euclidean distance between the results of the binary classification test (100% sensitivity and 100% specificity); and (ii) the maximum Euclidean distance between the results of the binary classification test (a 45° line). Sensitivity and specificity values, as well as the respective 95% confidence intervals (CIs), were calculated for each metabolite’s optimal cut-off value. All statistical analyses were performed using SAS software (version 9.4). The LC/MS profiles generated via LC–Q–TOF/MS were converted into mzML files using Hystar software (Bruker Technology), which were then uploaded to XCMS Online (www.xcmsonline.scripps.edu) [28]. All LC/MS profiles were processed using the “center-wave” algorithm with an allowance of 10 ppm on the experimental *m*/*z* and a minimum S/N ratio of 3 to extract the molecular features. The orbiwarp algorithm was used for the retention time correction, with a step size of 0.5 *m*/*z* and a maximum of 5 s allowed for deviation of the retention time.

## Figures and Tables

**Table 1:** Breast cancer patients and healthy controls demographic data.

Characteristic *	Breast Cancers (*n* = 23)	Healthy Controls (*n* = 35)	*p*-Value ^#^
Mean age range (years)	47.52 ± 9.79	42.00 ± 13.83	0.1028
Menopause status			0.7072
Premenopause	14 (60.87)	23 (65.71)	
Menopause	9 (39.13)	12 (34.29)	
Tobacco use			0.2019
No	19 (82.61)	33 (94.29)	
Yes	4 (17.39)	2 (5.71)	
Use of medication			0.2446
No	16 (69.57)	19 (54.29)	
Yes	7 (30.43)	16 (45.71)	
Childbearing			0.2663
No	8 (36.36)	18 (51.43)	
Yes	14 (63.64)	17 (48.57)	

* Values expressed in median ± standard deviation or frequency (%). Additional baseline characteristics are listed in Table S1 in the Supplementary Materials, ^#^ *p*-value by Student’s *t* test and Chi-squared/Fisher exact test.

**Table 2:** Breast cancer cases clinical and pathological characteristics.

Characteristic *	Breast Cancer Cases (*n* = 23)
**Grade**	
Grade 1	2
Grade 2	13
Grade 3	8
**Node status**	
Node negative	13
Node positive	10
**Stage**	
Stage 1	2
Stage 2	12
Stage 3	5
Stage 4	4
**ER status**	
ER positivity ≥ 10%	14
ER positivity < 10%	9
PR status	
PR positivity ≥ 10%	13
PR positivity < 10%	10
**HER2 status**	
HER2 positive	11
HER2 negative	12
KI67 status	
KI67 ≤ 20%	9
KI67 > 20%	14
**Molecular profile**	
Luminal A-like	5
Luminal B HER2 negative-like	8
Luminal B HER2 positive-like	3
HER2 positive (non-luminal)-like	3
Triple negative	3

ER: estrogen receptor, PR: progesterone receptor, HER2: human epidermal growth factor receptor 2, Ki67: antigen KI-67, * Values expressed in median ± standard deviation or frequency (%). Additional baseline characteristics are listed in Table S1 in the Supplementary Materials. The bold is the name of different categories.

**Table 3:** Potential salivary metabolites of breast cancer.

-Metabolite Number	*m*/*z*	Retention Time	Fold Change ^(a)^	*p*-Value	Raw Formula	Putative METLIN Identification ^(b)^	Mw g/mole (Error in ppm)
1	380.22	3.0	1.8	0.03	C_24_H_29_NO_3_	Donazepil	379.2147 (1)
2	457.23	2.5	3.1	0.003	C_45_H_72_O_16_	Dioscin or Desglucomusenin	868.482 (1)
3	622.33	3.1	2.3	0.04	C_32_H_40_N_6_O_6_	Dilazep or H-Thr-Trp-Trp-(Ile/Leu)-OH or H-Pro-Arg-Arg-Arg-OH	604.3009 (1)
4	633.34	3.0	2.2	0.02	C_38_H_48_O_8_	Tetrahydrogambogic acid	632.3349 (1)
5	440.23	3.4	3.7	0.03	C_15_H_31_N_9_O_5_	H-Arg-Arg-Ser-OH	417.2448 (3)
6	442.24	3.1	2.1	0.01	C_18_H_31_N_7_O_6_	H-His-Lys-(Ala-Ser)-OH or (Gly-Thr)-OH	441.2335 (1)
7	543.23	54.8	2.2	0.03	C_26_H_31_N_5_O_5_S	H-Ala-Lys-Phe-Trp-OH or H-Gly-Lys-Thr-Ser-OH or H-Arg-Arg-Ser-Ser-OH	525.2045 (1)
8	585.31	15.7	3.1	0.02	C_26_H_42_N_8_O_6_	H-Phe-Ile-Gln-Arg-OH	562.3227 (2)
9	596.31	3.0	2.7	0.03	C_25_H_38_N_8_O_8_	H-Glu-Phe-Gln-Arg-OH or H-Ile-Lys-Gln-Trp-OH	578.2812 (1)
10	630.33	3.1	1.9	0.04	C_32_H_45_N_7_O_5_	H-Phe-Lys-Lys-Trp-OH or H-Phe-Gln-Arg-Tyr-OH	607.3482 (1)
11	644.31	3.0	3.0	0.02	C_34_H_38_N_6_O_6_	H-Phe-Phe-Gln-Trp-OH	626.2852 (1)
12	669.42	36.9	2.1	0.04	C_35_H_67_O_7_P	PA 32:1 (c)	630.4624 (5)
13	718.39	19.4	6.3	0.01	C_34_H_66_NO_10_P	PS 28:0 (c)	679.4423 (9)
14	817.46	2.4	3.5	0.04	C_40_H_75_O_12_P	PI 31:1 (c)	778.4996 (9)
15	874.49	2.5	2.9	0.03	C_46_H_78_NO_10_P	PS 40:6 (c)	835.5362 (7)
16	919.47	3.0	2.5	0.02	C_47_H_77_O_13_P	PI 38:7 (c)	880.5101 (1)
17	533.22	2.5	2.4	0.01	C_23_H_43_O_9_P	PG 14:2 (c)	494.2644 (1)

^(a)^ Increasing fold change between the group of patients and of controls; ^(b)^ Metabolites putatively annotated according to results in METLIN database.

**Table 4:** Diagnostic test accuracy measurements for salivary metabolites breast cancer.

Metabolites	AUC	CI 95%	Optimal Cutoff Value	Sensitivity	CI 95%	Specificity	CI 95%
PG14:2	0.7329	0.5962–0.8697	19,325.80	65.22%	42.77–83.62%	77.14%	59.86–89.58%
PA 32:1	0.5988	0.4319–0.7656	1915.30	60.87%	38.54–80.29%	60.00%	42.11–76.23%
PS 28:0	0.6273	0.4644–0.7902	25,012.90	47.83%	26.82–69.41%	88.57%	73.26–96.80%
PI 31:1	0.5876	0.4250–0.7502	37,403.88	43.48%	23.19–65.51%	82.86%	66.35–93.44%
PS 40:6	0.5950	0.4357–0.7544	12,784.24	56.52%	34.49–76.81%	57.14%	39.35–73.68%
PI 38:7	0.6609	0.5132–0.8085	1951.16	60.87%	38.54–80.29%	71.43%	53.70–85.36%
H-His-Lys-(Ala-Ser)-OH or (Gly-Thr)-OH	0.7280	0.5962–0.8597	6274.70	73.91%	55.97–91.86%	62.86%	46.85–78.86%
H-Phe-Phe-Gln-Trp-OH	0.7478	0.6113–0.8844	1961.38	73.91%	55.97–91.86%	74.29%	59.81–88.77%
H-Phe-Ile-Gln-Arg-OH	0.6795	0.5373–0.8218	2406.38	69.57%	50.76–88.37%	68.57%	53.19–83.95%
H-Arg-Arg-Ser-OH	0.6621	0.5137–0.8105	4842.48	73.91%	55.97–91.86%	51.43%	34.87–67.99%
H-Glu-Phe-Gln-Arg-OH or H-Ile-Lys-Gln-Trp-OH	0.6565	0.4961–0.8170	5926.30	60.87%	40.92–80.81%	80.00%	66.75–93.25%
H-Phe-Lys-Lys-Trp-OH or H-Phe-Gln-Arg-Tyr-OH	0.6783	0.5356–0.8209	6499.44	56.52%	36.26–76.78%	74.29%	59.81–88.77%
H-Ala-Lys-Phe-Trp_OH or H-Gly-Lys-Thr-Ser-OH or H-Arg-Arg-Ser-Ser-OH	0.6497	0.5028–0.7966	3266.77	60.87%	40.92–80.81%	62.86%	46.85–78.86%
*m*/*z* 602.96	0.6584	0.5005–0.8163	6006.47	56.52%	36.26–76.78%	71.43%	56.46–86.39%
*m*/*z* 749.4	0.6696	0.5186–0.8205	3542.90	65.22%	45.75–84.68%	65.71%	49.99–81.44%
*m*/*z* 456.72	0.7441	0.6080–0.8802	101,578.83	69.57%	50.76–88.37%	74.29%	59.81–88.77%
*m*/*z* 475.70	0.7391	0.5979–0.8803	7027.85	65.22%	45.75–84.68%	74.29%	59.81–8877%
*m*/*z* 467.71	0.7702	0.6473–0.8931	8138.34	73.91%	55.97–91.86%	65.71%	49.99–81.44%
*m*/*z* 614.32	0.6665	0.5135–0.8194	11,030.99	52.17%	31.76–72.59%	77.14%	63.23–91.05%
*m*/*z* 457.73	0.6497	0.5132–0.8085	11,204.51	60.87%	40.92–80.81%	60.00%	43.77–76.23%
*m*/*z* 356.69	0.6845	0.5384–0.8306	2956.67	47.83%	27.41–68.24%	82.86%	70.37–95.34%
*m*/*z* 467.71	0.6857	0.5452–0.8262	12,496.76	73.91%	55.97–91.86%	60.00%	43.77–76.23%
*m*/*z* 534.23	0.6826	0.5389–0.8263	3642.90	65.22%	45.75–84.68%	65.71%	49.99–81.44%
*m*/*z* 1015.04	0.6609	0.5103–0.8114	9239.95	65.22%	45.75–84.68%	68.57%	53.19–83.95%
*m*/*z* 413.22	0.6783	0.5365–0.8200	2335.12	73.91%	55.97–91.86%	57.14%	40.75–73.54%
*m*/*z* 602.32	0.6646	0.5182–0.8110	4543.59	60.87%	40.92–80.81%	65.71%	49.99–81.44%
*m*/*z* 661.34	0.7025	0.5670–0.8380	1690.58	69.57%	50.76–88.37%	65.71%	49.99–81.44%
*m*/*z* 875.49	0.5652	0.4024–0.7281	14,840.39	39.13%	19.19–59.08%	77.14%	63.23–91.05%
*m*/*z* 594.82	0.6857	0.4024–0.7281	619.06	86.96%	73.19–100.00%	51.43%	34.87–67.99%
*m*/*z* 614.82	0.8124	0.4024–0.7281	2749.30	82.61%	67.12–98.10%	77.14%	63.23–91.05%
*m*/*z* 818.47	0.6186	0.4622–0.7751	7969.76	60.87%	40.92–80.81%	62.86%	46.85–78.86%

AUC: area under curve, CI: confidence interval; *m*/*z* = mass-to-charge ratio; ROC: Receiver operating characteristic.

**Table 5:** Identified salivary metabolites and their pathways.

METLIN Identification	Related Pathway
H-Arg-Arg-Ser-OH	N/S
H-His-Lys-(Ala-Ser)-OH or (Gly-Thr)-OH	N/S
H-Ala-Lys-Phe-Trp-OH Or H-Gly-Lys-Thr-Ser-OH or H-Arg-Arg-Ser-Ser-OH	N/S
H-Phe-Ile-Gln-Arg-OH	N/S
H-Glu-Phe-Gln-Arg-OH or H-Ile-Lys-Gln-Trp-OH	N/S
H-Phe-Lys-Lys-Trp-OH or H-Phe-Gln-Arg-Tyr-OH	N/S
H-Phe-Phe-Gln-Trp-OH	N/S
PA 32:1	Triacylglycerol Biosynthesis; Cardiolipin biosynthesis; Glycerophospholipid metabolism
PS 28:0	Phosphatidylcholine biosynthesis, Phosphatidylethanolamine biosynthesis, Glycerophospholipid metabolism and Lipid metabolism pathway
PI 31:1	N/F
PS 40:6	Phosphatidylcholine biosynthesis, Phosphatidylethanolamine biosynthesis, Glycerophospholipid metabolism and Lipid metabolism pathway
PI 38:7	Lysolipid incorporation into ER pathway, Phosphatidylcholine biosynthesis, Phosphatidylethanolamine biosynthesis, Glycerophospholipid metabolism and Lipid metabolism pathway
PG 14:2	Glycerophospholipid metabolism

N/S: not searched, N/F: not found in the Human Metabolome Database (HMDB), PA: phosphatidic acid; PS: phosphatidylserine; PI: phosphatidylinositol, PG: phosphatidylglycerol.

## Data Availability

All data and materials are stored in the Laboratory of Oral Histopathology, Health Sciences Faculty, University of Brasília, Brasilia, Brazil, and in the Laboratory of Bioanalytical Sciences and Miniaturization (LSABM), UMR CBI 8231, ESPCI Paris, CNRS, PSL University, Paris, France.

## Supplementary Materials

The following are available online at https://www.mdpi.com/2218-1989/10/12/506/s1, Figure S1: Overlay of the LC/MS profiles (Total Ions Chromatograms) for BC patients (in dot lines) and healthy controls (in plain lines), Figure S2: Overlay of the LC/MS profiles (Total Ions Chromatograms) for BC patients before treatment (in dot lines) and after treatment (in plain lines), Table S1: Subjects characteristics, Table S2: Salivary metabolites previously reported with their corresponding molecular weight and *m*/*z* of their parent ion, Table S3: List of medications used by subjects, Table S4: Retention time and *m*/*z* value of the most intense corresponding ion of unidentified compounds statistically overexpressed in patients with breast cancer, Table S5: Patients and their treatment schedules.
