# Supplementary material for: Using an Untargeted Metabolomics Approach to Identify Salivary Metabolites in Women with Breast Cancer
Source: Metabolites. 2020 Dec 10;10(12):506. doi: 10.3390/metabo10120506 (PMC7763953; doi:10.3390/metabo10120506)

Supplemental Fig 1: Overlay of the LC/MS profiles (Total Ions Chromatograms) for BC patients (in dot lines) and healthy controls (in plain lines).

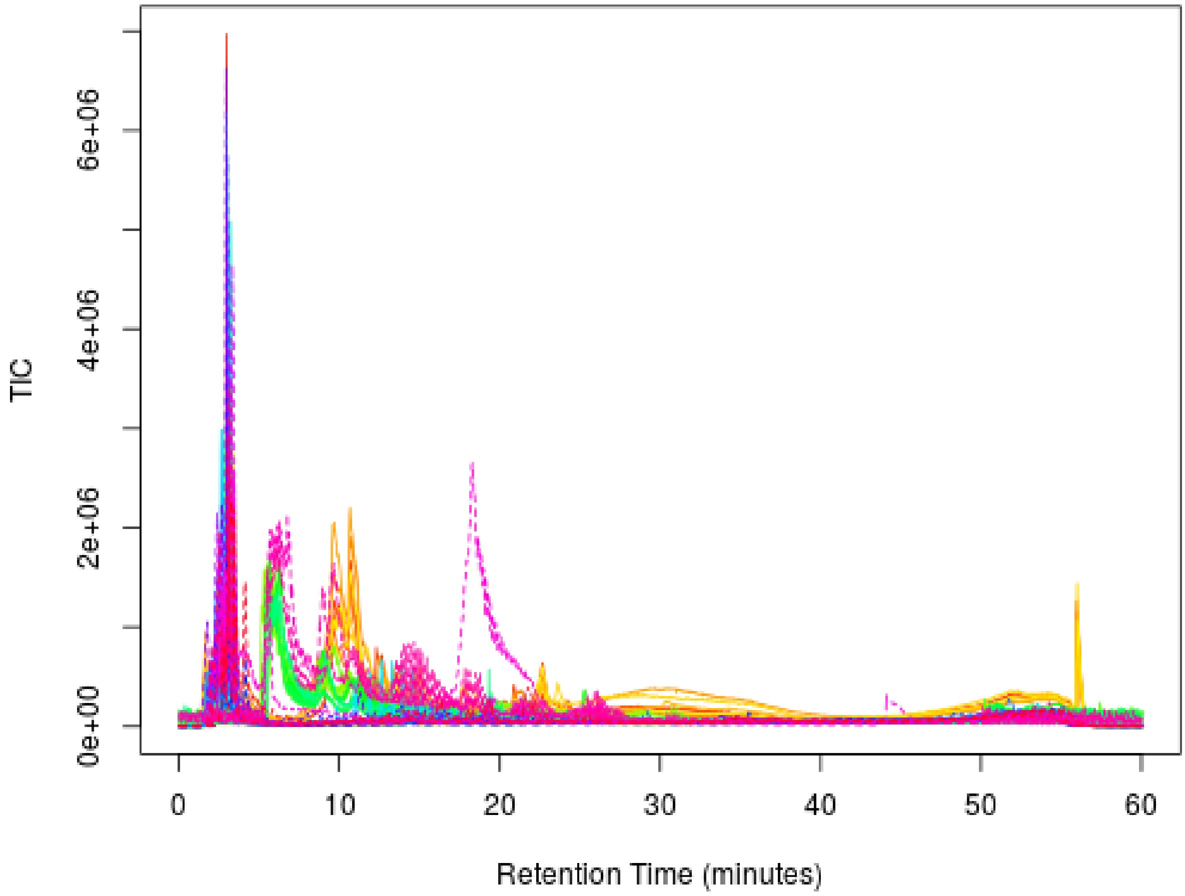

Supplement: Supplementary file 1 [file metabolites-10-00506-s001.zip › metabolites-1019029-supplementary/Supplemental Fig 1.pdf]
