# Supplementary material for: Using an Untargeted Metabolomics Approach to Identify Salivary Metabolites in Women with Breast Cancer
Source: Metabolites. 2020 Dec 10;10(12):506. doi: 10.3390/metabo10120506 (PMC7763953; doi:10.3390/metabo10120506)
Supplement: Supplementary file 1 [file metabolites-10-00506-s001.zip › metabolites-1019029-supplementary/Supplemental Table 1. Subjects charactheristics.pdf]

Supplementary Table 1. Subjects charactheristics

BC	Age	Menopause	Smoking	Hystological type	TNM	T	N	M	G	Mol subtype	ER	PR	Her2	Ki67	Metastasis site	Medication
BC1	34	no	yes	IDC + micropapillary component	IIA	T1c	N1	0	2	luminal A	60%	90%	neg	10%	no	no
BC2	42	no	no	IDC	IV	T4a	N1	1	3	luminal B HER2 +	95%	95%	3+	60%	node + bone + lung	no
BC3	51	yes	no	IDC	IIA	T2	N0	0	3	luminal B HER2-	0	10%	neg	90%	no	Losartan, Meformin, Indapamide
BC4	39	no	no	IDC	IIA	T2	N0	0	2	luminal A	80%	80%	neg	10%	no	no
BC5	42	no	yes	IDC	IA	T1c	N0	0	2	luminal A	90%	100 %	neg	20%	no	no
BC6	48	no	yes	IDC	IIA	T2	N0	0	1	luminal A	100%	1%	neg	3-5%	no	Losartan, Atenolol, Levothyroxine
BC7	56	yes	no	IDC	IIA	T2	N0	0	3	HER2+	0	0	3+	30%	no	Amlodipine, Levothyroxine
BC8	68	yes	no	IDC	IA	T1	N0	0	2	TN	0	0	neg	40%	no	no
BC9	42	yes	no	IDC	III B	T4	N1	0	2	luminal B HER2 +	70%	80%	3+	30%	no	no
BC10	37	no	no	IDC	IIB	T2	N1	0	2	luminal A	90%	80%	neg	20%	no	no
BC11	56	yes	yes	IDC	III B	T4	N1	0	2	luminal A	95%	80%	neg	20%	no	no
BC12	35	no	no	IDC	IIA	T2	N0	0	2	luminal B HER2-	100%	90%	neg	50%	no	no
BC13	53	yes	no	IDC	III B	T4b	N1	0	2	luminal B HER2 +	100%	90%	3+	40%	no	no
BC14	47	no	no	IDC	IIB	T2	N1	0	2	HER2+	neg	neg	3+	10%	no	Amlodipine, Hidroclorotiazide
BC15	71	yes	no	IDC	IV	T3	N0	1	2	luminal B HER2 +	90%	90%	3+	50%	lung	no
BC16	55	no	no	IDC	IIA	T2	N0	0	3	HER2+	neg	neg	3+	50%	no	Captopril
BC17	51	yes	no	IDC	IV	T4	N3	1	1	luminal A	70%	10%	neg	20%	bone	Atenolol

BC18	37	no	no	IDC	IIA	T2	N0	0	3	TN	neg	neg	neg	80%	no	no
BC19	51	no	no	IDC	III B	T4	N0	0	2	luminal B HER2 +	70%	neg	3+	25%	no	Atenolol
BC20	53	yes	no	IDC	III C	T4d	N3	0	3	TN	neg	neg	neg	30%	no	no
BC21	39	no	no	IDC	IIA	T2	N0	0	2	luminal B HER2 +	95%	97%	3+	45%	no	no
BC22	42	no	no	IDC	IV	T4	N1	1	3	HER2+	neg	neg	3+	70%	bone	no
BC23	44	no	no	IDC + squamous differentiation	IIB	T2	N0	0	3	HER2+	neg	neg	3+	50%	no	no

BC: breast cancer cases , IDC: invasive ductal carcinoma, TNM: tumor node metastasis stage, T: tumor, N: node, M: metastasis, G: tumor grade ; Mol: molecular, ER: estrogen receptor status, PR: Progeserone receptor status., HER2: human epidermal growth factor receptor 2, neg: negative; Ki 67: proliferation index.

HC	Age	Menopause	Smoking	Childbearing	Medication
HC1	28	no	no	0	Drospirenone, Etinilestradiol, Omeprazole
HC2	24	no	no	0	Mebeverine
HC3	35	no	no	1	no
HC4	29	no	no	0	no
HC5	26	no	no	0	Drospirenone, Etinilestradiol,
HC6	26	no	no	0	Drospirenone, Etinilestradiol,
HC7	22	no	no	0	no
HC8	25	no	yes	0	no
HC9	44	no	no	0	no
HC10	31	no	no	2	no
HC11	50	yes	no	0	Omeprazole
HC12	31	no	no	2	Levothyroxine
HC13	47	no	no	0	Levothyroxine
HC14	22	no	no	1	Drospirenone, etinilestradiol,
HC15	39	no	no	0	no
HC16	31	no	no	3	no
HC17	46	no	no	0	no

HC18	39	no	no	0	Metformnin
HC19	37	no	no	0	no
HC20	38	no	no	3	Metfottmin
HC21	73	yes	no	0	no
HC22	55	yes	no	4	no
HC23	39	no	no	2	no
HC24	37	no	no	0	Atorvastatin
HC25	74	yes	no	2	Losartan, levothyroxine, atorvastatin
HC26	44	no	no	3	no
HC27	41	no	no	1	no
HC28	55	yes	no	1	Anlodipina, Telmisartan
HC29	65	yes	no	2	Metformin, Sertraline, Etinilestradiol
HC30	54	yes	no	2	Venlafaxine, Etinilestradiol
HC31	48	yes	no	0	no
HC32	54	yes	no	1	Losartan, Hydrochlorothiazide, Atorvastatin
HC33	54	yes	no	0	no
HC34	56	yes	no	3	no
HC35	51	yes	yes	2	no

HC: healthy control cases
