# Supplementary material for: Using an Untargeted Metabolomics Approach to Identify Salivary Metabolites in Women with Breast Cancer
Source: Metabolites. 2020 Dec 10;10(12):506. doi: 10.3390/metabo10120506 (PMC7763953; doi:10.3390/metabo10120506)
Supplement: Supplementary file 1 [file metabolites-10-00506-s001.zip › metabolites-1019029-supplementary/Supplemental Table 2 Previously identified metabolites.pdf]

Supplemental Table 2. Salivary metabolites previously reported with their corresponding molecular weight and m/z of their parent ion.

Metabolites	Molecular Weight	m/z (M+H)
2-Hydroxypentanoate	117.124	118.12
Dodecanoic acid	200	201.37
N-Acetylneuraminate	309	310.26
N-ε-Acetyllysine	188.224	189.23
1,3-Diaminopropane	74.124	75.13
2-hydroxy-4 methylvaleric acid	132.159	133.16
2-Hydroxy-4-methylpentanoate	131.151	132.15
2-Hydroxybutanedioate	132.071	133.07
2-Hydroxybutyric acid	104.104	105.10
2-Hydroxyvaleric acid	118.131	119.13
2-Ketobutyric acid	102.088	103.09
3-Hydroxybutyric acid	104.104	105.10
4- Methilbenzoate	135.142	136.14
4-Hydroxyphenylpyruvic acid	180.157	181.16
4-methoxyphenylacetic acid	166.173	167.17
4-Trimethylammoniobutanoic acid	146.207	147.21
5-Aminolevulinic acid	131.129	132.13
6,N6,N6-Trimethyl-L-lysine	188.267	189.27
7-Methylguanine	165.152	166.15
Adenosine	267.241	268,24
Alanyl-Alanine	160.171	161.17
alpha-Ketoisovaleric acid	116.115	117.12
Arachidic acid	312.530	313.53
arginine	174.201	175.20
Aspartic acid	133.102	133.10
beta-alanine	89.093	90.09
betaine	117.146	118.15
Butyric acid	88.105	89.11
c-aminobutyric acid	103.119	104.12

cadaverine	102.178	103.18
Caproic acid	116.158	117.16
choline	104.170	105.17
cis-Aconitate	174.108	175.11
Citric acid	192.123	193.12
citrulline	175.185	176.19
Cystine	240.3	241.30
Cytosine	111.102	112.10
D- alpha-aminobutyric acid	103.119	104.12
Desaminotyrosine	166.173	167.17
diaminopropane	74.124	75.13
Diglycine	132.119	133.12
Ethanolamine	61.083	62.08
Ethanolamine Phosphate	141.063	142.06
formic acid	46.025	47.03
glutamic acid	147.129	148.13
glutamine	146.144	147.14
Glutathione	307.323	308.32
glycine	75.066	76.07
glycolic acid	76.051	77.05
Glycyl-L-leucine	188.22	189.22
Guanine	151.126	152.13
Guanosine	283.240	284.24
heptanoic acid	130.184	131.18
histidine	155.154	156,15
homocysteine	135.185	136.19
Hydrocinnamic acid	150.174	151.17
Hydroxylysine	162.187	163.19
Hypotaurine	109.147	110.15
Hypoxanthine	136.111	137.11
Inosine	268.226	269.23
isoleucine	131.172	132.17
Isopropanolamine	75.110	76.11

L- alpha-aminobutyric acid	103.119	104.12
L-Alanine	89.093	90.09
L-carnitine	161.198	162.20
lactic acid	90.077	91.08
leucine	131.172	132.17
Leucinic acid	132.157	133.16
lysine	146.187	147.19
LysoPC (16:0)	495.630	496.63
LysoPC (18:1)	521.676	522.68
LysoPC (22:6)	567.694	568.69
LysoPE (18:2/0:0)	477.571	478.57
methionine	149.211	150.21
Myristic acid	228.370	229.37
N-Acetyl-L-phenylalanine	207.225	208.23
N-Acetylornithine	174.197	175.20
N.N-Dimethylglycin	103.121	104.12
N1 –acetylspermidine	187.282	188.28
N1-acetyl-spermine	244.376	245,38
N1-acetylputrescine	130.188	131.19
N6-Acetyl-L-lysine	188.224	189.22
N8-Acetylspermidine	187.282	188.28
Nicotinic acid	123.109	124.11
Octanoate	143.206	144.21
octanoic acid	256.424	257.42
ornithine	132.161	133.16
p-hydroxyphenylacetic acid	152.147	153.15
palmitic amid	284.477	285.48
phenylalanine	165.189	166.19
Phenyllactic acid	166.173	167.17
Phosphoserine	185.072	186.07
Pipecolic acid	129.157	130.16
piperidine	85.147	86.15

proline	115.130	116.13
putrescine	88.151	89.15
Pyrroline hydroxycarboxylic acid	129.114	130.11
Ribose 5-phosphate	230.110	231.11
saccharic acid derivative (N-Acetylneuraminic acid)	210.138	211.14
serine	105.092	106.09
spermidine	145.245	146.25
spermine	202.340	203.34
sphingolipid (phytosphingosine)	317.514	318.51
taurine	125.147	126.15
terephthalic acid	166.130	167.13
threonine	119.119	120.12
Trimethylamine	59.110	60.11
Tryptophan	204.225	205.23
tyrosine	181.188	182.19
urea	60.055	61.06
Ureidosuccinic acid	176.127	177.13
uric acid	168.110	169.11
valine	117.146	118.15
