# Supplementary material for: Using an Untargeted Metabolomics Approach to Identify Salivary Metabolites in Women with Breast Cancer
Source: Metabolites. 2020 Dec 10;10(12):506. doi: 10.3390/metabo10120506 (PMC7763953; doi:10.3390/metabo10120506)
Supplement: Supplementary file 1 [file metabolites-10-00506-s001.zip › metabolites-1019029-supplementary/Supplemental Table 3. list of medications.pdf]

Supplement Table 3. List of medications used by subjects

Medications	Molecular Mass	m/z DA	Number of cases using	Number of controls using
Losartan	422.91 g/mol	423.91	2	2
Metformin	129.16364 g/mol	130.16	1	2
Indapamide	365.835 g/mol	366.83	1	0
Mebeverine	429.6 g/mol	430.6	0	1
Sertraline	306.229 g/mol	307.22	0	1
Clonazepam	315.715 g ·mol	316.71	0	0
Valsartan	435.519 g/mol	436.51	0	0
Levothyroxine	776.874 g/mol	777.87	2	3
Amlodipine	408.879 g/mol	409.87	2	1
Hydrochlorothiazide	297.74 g/mol	298.74	1	1
Captopril	217.29 g/mol	218.29	1	0
Atenolol	266.336 g/mo	267.33	3	0
Drospirenone	366.493 g/mol	367.49	0	1
Omeprazole	345.42 g/mol	346.42	0	2
Venlafaxine	277.402 g/mol	278.40	0	1
Telmisartan	514.617 g/mol	515.61	0	1
Ethinylestradiol	296.403 g/mol	297.40	0	3
