# Supplementary material for: Using an Untargeted Metabolomics Approach to Identify Salivary Metabolites in Women with Breast Cancer
Source: Metabolites. 2020 Dec 10;10(12):506. doi: 10.3390/metabo10120506 (PMC7763953; doi:10.3390/metabo10120506)
Supplement: Supplementary file 1 [file metabolites-10-00506-s001.zip › metabolites-1019029-supplementary/supplemental Table 4 Unidentified metabolites.pdf]

Supplemental Table 4. Retention time and m/z value of the most intense corresponding ion of unidentified compounds statistically overexpressed in patients with breast cancer

m/z	Retention time	fold change (a)	p-value
**356.69**	3.7	5.8	0.02
**413.22**	2.6	2.0	0.03
**456.72**	2.6	3.3	0.002
**457.73**	2.5	2.8	0.02
**467.20**	3.0	2.3	0.02
**467.71**	2.8	3.3	0.01
**475.70**	2.6	3.4	0.002
**534.23**	2.5	1.9	0.04
**594.82**	3.0	2.2	0.04
**602.32**	2.7	3.5	0.04
**602.96**	3.1	2.5	0.01
**614.32**	2.6	2.4	0.01
**614.82**	2.8	3.0	0.04
**661.34**	3.0	2.7	0.04
**749.4**	3.0	2.3	0.02
**818.47**	2.4	3.2	0.04
**875.49**	2.5	2.8	0.04
**1,015.04**	3.1	2.4	0.03

HPLC : high performance liquid chromatography; (a) Increasing fold change between the group of patients and of controls
