# Supplementary material for: Using an Untargeted Metabolomics Approach to Identify Salivary Metabolites in Women with Breast Cancer
Source: Metabolites. 2020 Dec 10;10(12):506. doi: 10.3390/metabo10120506 (PMC7763953; doi:10.3390/metabo10120506)
Supplement: Supplementary file 1 [file metabolites-10-00506-s001.zip › metabolites-1019029-supplementary/Supplemental Table 5. Patients and treatment charactheristics.pdf]

Supplemental Table 5. Patients and their treatment schedules.

Breast cancer cases	TNM	T	N	M	Grade	Molecular subtype	Treatment	Response
BC3	IIA	T2	N0	0	3	luminal B HER2-	NACT: AC x 4 cycles- paclitaxel	CR
BC9	IIA	T2	N0	0	3	HER2+	NACT: Docetaxel + carboplatin + trastuzumabe	PR
BC10	IA	T1	N0	0	2	TN	NACT: AC x 4 cycles- carboplatin and paclitaxel	PR
BC13	IIIB	T4	N1	0	2	luminal A	NACT	PR
BC19	IIA	T2	N0	0	3	HER2+	NACT: AC x 4 cycles- paclitaxel+ pertuzumabe + trastuzumabe x 4 cycles	CR
BC20	IV	T4	N3	1	1	luminal A	Paliative tamoxifen	PR
BC21	IIA	T2	N0	0	3	TN	NACT: AC x 4 - paclitaxel	CR
BC24	IIA	T2	N0	0	2	luminal B HER2 +	NACT: AC x 4 cycles- Docetaxel + pertuzumabe + trastuzumabe	CR
BC25	IV	T4	N1	1	3	HER2+	Paliative treatment: Docetaxel + Pertuzumab+ trastuzumab 6 cycles	PR
BC26	IIB	T2	N0	0	3	HER2+	NACT: AC x 4 cycles- paclitaxel+ pertuzumabe + trastuzumabe x 4 cycles	CR

TNM: Tumor, Node Metastasis; NACT: neo-adjuvant chemotherapy, AC: doxorubicin plus cyclophosphamide, CR: complete response, PR: partial response, TN: triple negative.
